# A machine learning approach for predicting suicidal thoughts and behaviours among college students

**DOI:** 10.1038/s41598-021-90728-z

**Published:** 2021-06-15

**Authors:** Melissa Macalli, Marie Navarro, Massimiliano Orri, Marie Tournier, Rodolphe Thiébaut, Sylvana M. Côté, Christophe Tzourio

**Affiliations:** 1 Inserm, Bordeaux Population Health Research Center, UMR 1219, University of Bordeaux, 146 rue Léo Saignat, 33076 Bordeaux Cedex, Bordeaux France; 2 McGill Group for Suicide Studies, Douglas Mental Health University Institute and Department of Psychiatry, McGill University, Montreal, QC Canada; 3 Charles Perrens Hospital, 33000 Bordeaux, France; 4 Inria SISTM, 33000 Bordeaux, France; 5 CHU de Bordeaux, 33000 Bordeaux, France; 6 School of Public Health, University of Montreal, Montreal, QC H3T 1J4 Canada

**Keywords:** Psychology, Risk factors

## Abstract

Suicidal thoughts and behaviours are prevalent among college students. Yet little is known about screening tools to identify students at higher risk. We aimed to develop a risk algorithm to identify the main predictors of suicidal thoughts and behaviours among college students within one-year of baseline assessment. We used data collected in 2013–2019 from the French i-Share cohort, a longitudinal population-based study including 5066 volunteer students. To predict suicidal thoughts and behaviours at follow-up, we used random forests models with 70 potential predictors measured at baseline, including sociodemographic and familial characteristics, mental health and substance use. Model performance was measured using the area under the receiver operating curve (AUC), sensitivity, and positive predictive value. At follow-up, 17.4% of girls and 16.8% of boys reported suicidal thoughts and behaviours. The models achieved good predictive performance: AUC, 0.8; sensitivity, 79% for girls, 81% for boys; and positive predictive value, 40% for girls and 36% for boys. Among the 70 potential predictors, four showed the highest predictive power: 12-month suicidal thoughts, trait anxiety, depression symptoms, and self-esteem. We identified a parsimonious set of mental health indicators that accurately predicted one-year suicidal thoughts and behaviours in a community sample of college students.

## Introduction

College students are vulnerable to mental health problems and suicidal thoughts and behaviours (STB)^1,2^. In a large study in eight countries the 12-month prevalence rates were 17.2% for suicidal ideation, 8.8% for suicidal planning, and 1.0%, for suicide attempt^3^. Factors that may contribute to the increased risk of STB in this population include the transition from high-school to university, increasing workload, increased psychosocial stress and academic pressures, and adaptation to a new environment^4^. Avoiding the onset or aggravation of STB requires early detection of students at risk, to help them access mental health services or having them engaged in coaching strategies^5,6^. However, identifying students with STB is challenging due to limited resources on campus^7^, and because college students may be reluctant to share information about their mental health. Effective screening would require (1) the identification of characteristics that predict STB; and (2) minimally intrusive questions integrated into short assessments easier to administer to large populations. Most previous studies of STB prediction in students have been based on logistic regression models that account for a limited number of predictors, and have provided association measures^8,9^. However, the identification of factors associated with STB does not necessarily imply that they could help predict future STB^10,11^ and for that, models specifically designed for prediction are needed. Moreover, some variables used in previous studies (e.g., psychiatric assessment)^12^ are impractical to assess in a large population of students as they require the expertise of a trained clinician. As pointed out in a recent paper summarizing 50 years of research on STB, further research should shift from identification of risk factors associated with STB to focus on developing predictive algorithms using machine learning methods^13^. Such methods enable the inclusion of several risk and protective factors, while accounting for their potential interactions^14,15^, which is consistent with the shared concept that STB result from complex interactions between social, psychiatric, psychological, and environmental factors^16^.

In this study we applied a machine learning method to develop an algorithm to predict STB in the next 12 months after baseline assessment using a large longitudinal cohort of French university students. All analyses were stratified by gender as recommended^16–18^.

## Methods

### Study design and participants

Our study sample comprised participants in the ongoing internet-based Students’ Health Research Enterprise (i-Share) project—a prospective population-based study on students’ health which was launched in some French universities in 2013. Students were informed about the purpose and aims of the study through flyers, communications in classes or social media. To be eligible students must be registered at a university or higher education institute, be at least 18 years of age, and be able to read and understand French. Volunteers provided an on-lined informed consent. The enrolment procedure has been previously described^19^.

The i-Share protocol was approved by the “Commission nationale de l’informatique et des libertés” (CNILNational Commission of Informatics and Liberties) (number: DR-2013-019), which ensures that data collection does not violate freedom, rights, or human privacy. The study follows the principles of the Declaration of Helsinki and the collection, storage and analysis of the data comply with the General Data Protection Regulation (EU GDPR). At enrolment (i.e., baseline assessment), self-administered on-line questionnaires collected sociodemographic characteristics, physical and mental health parameters, personal and familial history, living conditions, lifestyle habits, and substance use. One year later, students were invited by email to complete a follow-up questionnaire. Three reminder emails were sent at 14, 28, and 33 days following the invitation. For the present longitudinal study, we used data from a sample of students who were included in the i-Share cohort study between February 2013 and September 2019, who participated in the follow-up, and for whom data on STB were available.

Baseline information was available for 15, 667 students. These students were solicited to participate in the follow-up, and 5255 agreed to participate (33.5% response rate). At baseline, compared to the students who participated in the follow-up, the non-participants reported slightly more 12-month suicidal ideation (n = 2285, 22.0% vs. n = 1151, 21.9%; *p* = 0.0004) and more lifetime suicide attempts (n = 682, 6.6% vs. n = 298, 5.7%; *p* = 0.0004). Additionally, the non-respondents were more likely boys (n = 2963, 25.9% vs. n = 1099, 20.9%; *p* < 0.0001). We did not observed differences between participants and non-participants for the year of study or parental depression history (Supplementary Table 1). Among the respondents, 189 (3.6%) were excluded because they did not answer the STB-related questions.

### Measures

The one-year follow-up questionnaire included questions about suicidal thoughts and suicide attempts during the last 12 months. Participants who reported having occasional or frequent suicidal thoughts and/or suicide attempts were coded as positive for STB.

We considered baseline assessments of 70 potential predictors (Supplementary Table 2). These variables included socio-demographic characteristics (e.g., age, year of study, scholarship, and accommodation type), lifestyle habits (e.g., time spent on screens and sleep quality), familial characteristics (e.g., perceived parental support, parental divorce, and parental history of depression), physical health (e.g., handicap and perceived health), and substance use (e.g., tobacco and alcohol use). Baseline characteristics also included history of diagnosed psychiatric disorders, lifetime suicide attempts and suicidal thoughts during the 12 months preceding inclusion (latter called baseline STB). We measured several mental health parameters with validated scales: depression symptoms using the 9-item Patient Health Questionnaire (PHQ-9)^20^; trait anxiety using the Spielberger State-Trait Anxiety Inventory (STAI-YB)^21^; self-esteem using the Rosenberg scale^22^; perceived stress using the Perceived Stress Scale (PSS-4)^23^; and impulsivity using the Barratt Impulsivity Scale (BIS-11)^24^.

Childhood adversities are not investigated in the baseline questionnaire. In order to take into account these important potential predictors in our models, a subsample of 1911 participants was administered a supplementary questionnaire adapted from the Childhood trauma questionnaire^25^. This questionnaire included 17 variables assessing experiences of sexual abuse, physical or psychological maltreatment, or neglect (Supplementary Table 2).

### Statistical analyses

We first described the overall study sample and according to the gender. Continuous variables are expressed as mean ± standard error. Categorical variables are described as the proportion.

#### Prediction of one-year STB

To predict STB we used a random forests model, which is a non-parametric ensemble machine learning method applicable for both classification and regression prediction^26^. This technique is broadly used due to its high performance and robustness, and because it enables the use of variables independently of type and distribution^27^. Random forests are based on the aggregation of a set of decision trees created through recursive bootstraps of the initial sample^28^. In each bootstrap sample, a decision tree is created using two-third of the observations. The remaining one-third, termed the out-of-bag sample, is used to obtain an unbiased performance measure of the created algorithm. This evaluation of prediction performance yields a measure termed the out-of-bag error, which represents the overall error of the algorithm in terms of outcome prediction. The out-of-bag sample is also used to calculate the relative importance of each variable for the prediction. To this end, the value of a given variable is randomly shifted in the out-of-bag sample, and any resulting change of the out-of-bag error reflects the variable’s importance in the prediction. Finally, all individual decision trees are aggregated to create the final predictor algorithm. To carry out these analyses we used the *randomForest* and *caret* packages in SAS and R. Missing data on the predictors (2%) were handled using the R *missForest* algorithm^29^ specifically designed to deal with missing data in random forest models.

#### Predictors

For the main analyses, the 70 potential predictors were included in the model. We then performed two secondary analyses. First, we re-estimated our models in a subsample of participants who did not report STB at baseline to better identify new cases^30^. Second, we re-estimated our models in the subsample including data on childhood adversity.

#### Evaluation of model performance

We evaluated the prediction quality of our models in the testing sample using the following performance metrics: (1) out-of-bag error, obtained using the out-of-bag sample of the training set, which represents the overall error in the prediction (ranges from 0%, indicating that no individual is correctly classified, to 100%, indicating that all individuals are correctly classified); (2) area under the curve (AUC)^31^, which measures the accuracy of discrimination performance represented by the predicted true positive rate against the false positive rate (ranges from 0.5, indicating prediction by chance, to 1, indicating perfect prediction); (3) sensitivity, representing the rate of actual cases (i.e. students reporting STB) identified by the algorithm; and (4) the positive predictive value, describing the proportion of algorithm-predicted cases that are actual cases. To prevent these performances to be over-fitted and to increase the generalizability of the prediction model, we estimated these indices through cross-validation. We therefore split randomly the initial dataset into 10-folds, we created the model using 9 of the 10 folds and tested on the remaining fold. We repeated this process until all the folds were used as test sets. All the values needed for the prediction, i.e. the real outcome, predicted outcome and probabilities of belonging in each class of the outcome, were calculated in each test sample and stored in an independent file; the final prediction metrics were then obtained with all the stored values and we reported the mean value of the out-of-bag error across the 10 models.

All models were carried out in accordance with the Transparent Reporting of a Multivariable Prediction Model for Individual Prognosis or Diagnosis (TRIPOD) statement for prediction model development^32^.

## Results

### Description of the sample

The final study population comprised 5066 students, including 4005 (79.1%) girls and 1061 (20.9%) boys. Of the 5066 participants, 874 (17.3%) students reported experiencing STB in the past 12 months (17.1% reported suicidal ideation and 0.7% suicide attempts). The STB prevalence did not significantly differ between girls (n = 696; 17.4%) and boys (n = 178; 16.8%). Among the 874 students who reported STB, 61.3% (n = 536) reported 12-month suicidal thoughts (with or without history of lifetime suicide attempts), and 14.6% (n = 128) reported a lifetime suicide attempt at baseline.

The main baseline characteristics did not significantly differ according to gender (Table 1). The mean participant age was 20.7 years (SD 2.6). Over one-third of the sample (n = 1932; 38.1%) was in their first year of university education. The majority of the students lived alone in an apartment (n = 1544; 30.5%) or at their parents’ home (n = 1495; 29.5%), and 17.5% (n = 884) described their current economic situation as difficult or very difficult. The most prevalent indicators of childhood adversity were maternal depression history (n = 1536; 30.3%) and parental divorce or separation (n = 1484; 29.3%). At baseline, one in five students reported 12-month suicidal thoughts (n = 1072; 21.2%) and 5.4% (n = 275) reported a lifetime suicide attempt.

**Table 1:** Sample characteristics at baseline.

	Total sample (n = 5066)	Girls (n = 4005)	Boys (n = 1061)
Age in years, mean (SD)	20.7 (2.6)	20.7 (2.6)	21.2 (2.7)
**Accommodation type**			
At parents’ home	1496 (29.5)	1166 (29.1)	330 (31.1)
University residence	569 (11.2)	442 (11.0)	127 (12.0)
In apartment, couple, colocation	1276 (25.2)	1028 (25.7)	248 (23.4)
In apartment, alone	1544 (30.5)	1216 (30.3)	328 (30.9)
Missing	181 (3.6)	153 (3.8)	28 (2.6)
**Opinion on resources**			
Satisfactory to very satisfactory	4182 (82.6)	3282 (82.0)	900 (84.8)
Unsatisfactory to totally unsatisfactory	884 (17.5)	723 (18.1)	161 (15.2)
**Year of study**			
First year	1932 (38.1)	1602 (40.0)	330 (31.1)
Second year and higher	3134 (61.9)	2403 (60.0)	731 (68.9)
**Perceived parental support in childhood**			
Moderate to very high	4623 (91.3)	3361 (91.4)	962 (90.7)
Low to none	386 (7.6)	304 (7.6)	82 (7.7)
Missing	57 (1.1)	40 (1.0)	17 (1.6)
**Parental divorce**			
Yes	1484 (29.3)	1192 (29.8)	292 (27.5)
No	3447 (68.0)	2708 (67.6)	739 (69.7)
Missing	135 (2.7)	105 (2.6)	30 (2.8)
**Paternal depression history**			
Yes	856 (16.9)	719 (18.0)	137 (12.9)
No	3536 (69.8)	2758 (68.9)	778 (73.3)
Missing	674 (13.3)	528 (13.2)	146 (13.8)
**Maternal depression history**			
Yes	1536 (30.3)	1256 (31.4)	280 (26.4)
No	3010 (59.4)	2346 (58.6)	664 (62.6)
Missing	520 (10.3)	403 (10.1)	117 (11.0)
**12-month suicidal ideation**			
Yes	1072 (21.2)	850 (21.2)	222 (20.9)
No	3856 (76.1)	3038 (75.9)	818 (77.1)
Missing	138 (2.7)	117 (2.9)	21 (2.0)
**Lifetime suicide attempts**			
Yes	275 (5.4)	228 (5.7)	47 (4.4)
No	4696 (92.7)	3695 (92.3)	1001 (94.3)
Missing	95 (1.9)	82 (2.1)	13 (1.2)

All data presented as n (%) unless otherwise noted.

### Prediction of suicidal thoughts and behaviours

**Figure 1:**
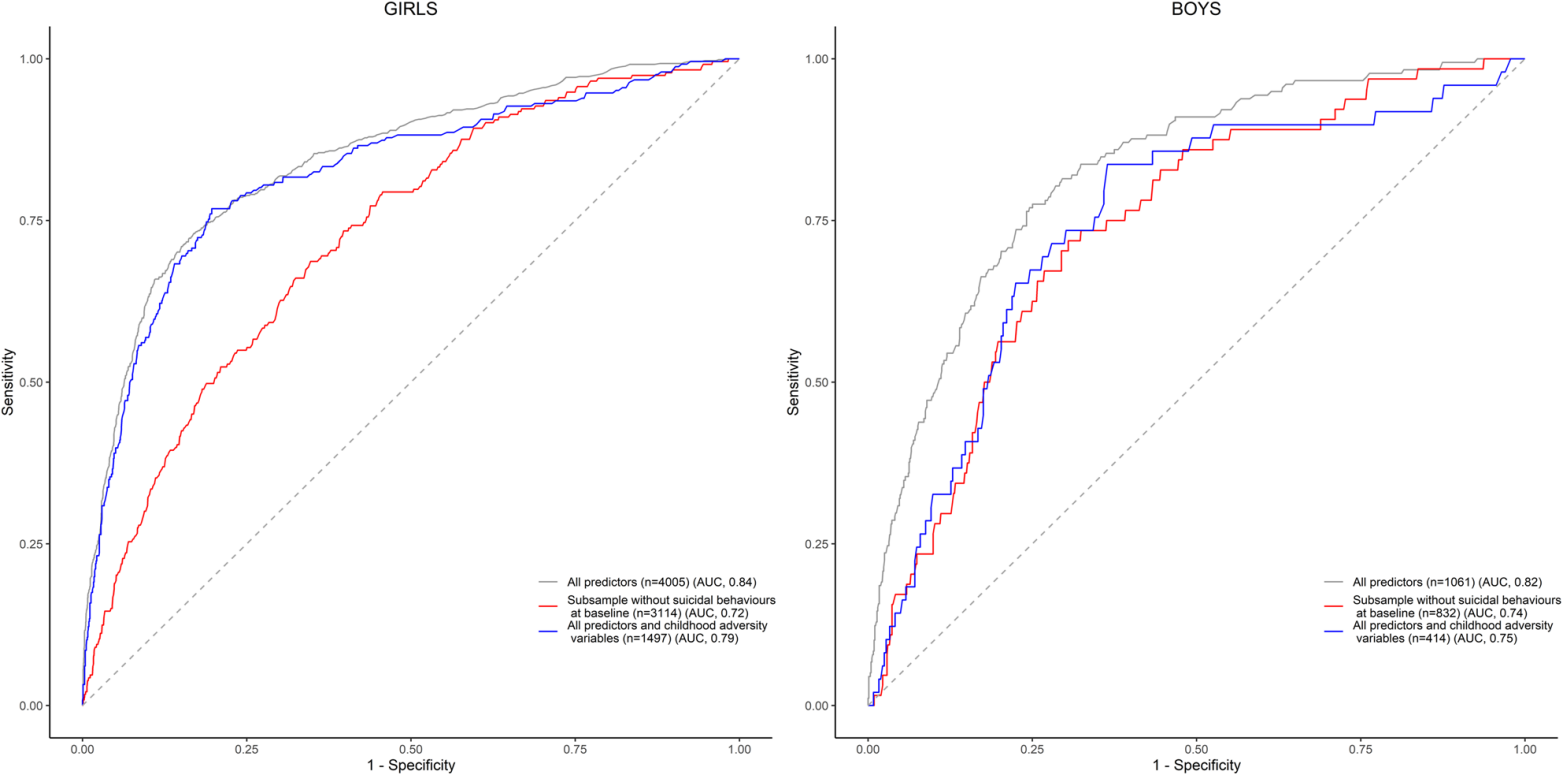
Area-under-the-curve plots of the sensitivity and specificity of random forests predictive models for suicidal thoughts and behaviours, stratified by gender.

**Figure 2:**
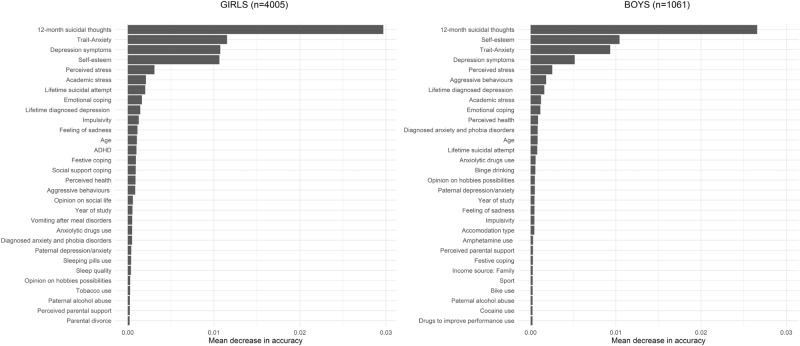
Ranking of the importance of baseline variables in a random forests model for predicting one-year suicidal thoughts and behaviours, stratified by gender.

Among girls, the predictive model had an out-of-bag error of 24.6%, suggesting the overall misclassification of a quarter of the female participants. Among boys, the out-of-bag error was 28.1%. The model showed an AUC of 0.84 (95% CI 0.83–0.86) for girls, indicating a discrimination 68% better than chance, and 0.82 (95% CI 0.79–0.86) for boys (Fig. 1). The sensitivity was 0.79 for girls and 0.81 for boys, indicating that the model correctly predicted 79–81% of the actual cases (Table 2). The predictive positive values were 0.40 and 0.36 for girls and boys, respectively, meaning that 40% and 36% of predicted cases were actually cases. Analysis of the variables’ importance for the prediction, as measured by the mean decrease in accuracy, revealed that the following four variables were the most predictive in both girls and boys: 12-month suicidal thoughts at baseline, self-esteem, trait anxiety, and depression symptoms (Fig. 2).

**Table 2:** Predictive performances metrics.

	Girls			Boys		
	All predictors	All predictors in a subsample without participants with baseline suicidal behaviours	All predictors and childhood adversity variables	All predictors	All predictors in a subsample without participants with baseline suicidal behaviours	All predictors and childhood adversity variables
Out-of-Bag error (%)	24.6	31.3	25.2	28.1	25.6	28.6
Area under the curve (95% Confidence Interval)	0.84 (0.83;0.86)	0.72 (0.70;0.76)	0.82 (0.79;0.86)	0.82 (0.79;0.86)	0.74 (0.68;0.80)	0.75 (0.67;0.83)
Sensitivity	0.79	0.63	0.79	0.81	0.62	0.76
Positive predicted value	0.40	0.14	0.37	0.36	0.17	0.22

### Secondary analyses

**Figure 3:**
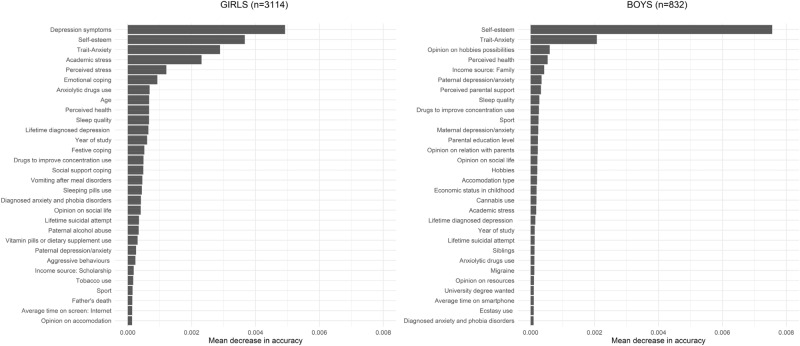
Ranking of the importance of baseline variables in a random forests model for predicting one-year suicidal thoughts and behaviours, removing participants with baseline suicidal thoughts and behaviours, stratified by gender.

We repeated these analyses in a subsample of participants who did not report STB at baseline, and found that the predictive performances were lower than in the main analyses. For girls (n = 3114) and boys (n = 832), respectively, the AUC was 0.72 and 0.74, and the sensitivity was 0.63 and 0.62. Variable importance for the prediction was different between girls and boys, with the following main predictive variables for girls: depression symptoms, self-esteem, trait anxiety, and academic stress (Fig. 3). For boys, we found one main predictor i.e. self-esteem followed by trait anxiety (Fig. 3). We then fitted our random forests models among the 1497 girls and 414 boys who answered the childhood adversity questionnaire. The predictive performances were similar for girls (AUC 0.82; sensitivity of 79%) and boys (AUC 0.75; sensitivity of 76%). In girls, the four main predictive variables were baseline suicidal thoughts, depression symptoms, self-esteem, and trait anxiety. In boys, the four top predictors were 12-month suicidal thoughts, perceived stress, trait anxiety and self-esteem (Supplementary Figure 1). Thus, in both genders, childhood adversity variables did not contribute to STB prediction.

## Discussion

Using random forests models in this large sample of college students we found that four main baseline variables predicted STB at 12-month: suicidal thoughts at baseline, trait anxiety, depression symptoms, and self-esteem. The model including these variables showed good predictive performance (AUC = 0.8) estimated using cross-validation. In secondary analyses in a subsample excluding participants who reported STB at baseline, the main predicting variables were depressive symptoms, self-esteem, and academic stress for girls and mainly self-esteem for boys. These predictors differ according to gender only among participants who did not report STB at baseline. Finally, childhood adversity variables did not contribute to STB prediction.

To our knowledge, only two prior studies have developed STB predictive models in students and reported comparable predictive performances to our study. One study used the random forests method to predict suicide attempts among medical students, using a cross-sectional design^33^. The other study used a logistic regression model to develop a risk-screening algorithm for persistence of suicidal behaviours during college^34^.

STB prediction was not influenced by childhood trauma or perceived parental support, which are usually strongly associated with STB in young adults^35,36^. These results are in line with previous studies^34,37^. This finding highlights that association does not necessarily means prediction^11^, and that proximal risk factors of STB may be better than distal or early life one for predicting one-year STB^38^. We can also assume that important predictors such as depression symptoms are the downstream consequences of higher adversity during childhood, and as they are more recent, they could be overshadowing the importance of early adversity in STB prediction. Furthermore, following the diathesis stress model of suicide, the predictors we found (anxiety, depression) might affect more vulnerable individuals who have experienced childhood adversities^39^.

We identified a small number of major predictors that ensured high accuracy in STB prediction. These predictors, derived from short and commonly used questionnaires, may help developing a large-scale screening tool for university students. For example, they could be integrated into a short online screening administered upon college entrance. An online questionnaire may prove acceptable to students, and would provide an alternative to mental health assessment by a physician for students who are often reluctant to disclose sensitive personal information in face-to-face interviews^40,41^.

The quantitatively most important predictor was suicidal thoughts at baseline^34,42^. Likewise, anxiety and depression were often comorbid with STB in students^43^. Interestingly, self-esteem emerged as one of the main predictors of STB. Low self-esteem is known to be a part of social anxiety, and to overlap with depression, both of which are associated with STB^44^. Self-esteem, which is an important marker of psychological vulnerability in young adults^45–47^ has also been found associated with suicidality^48^. Our study showed that self-esteem is an independent and prominent predictive marker of STB and should therefore be used in a screening tool.

Overall, our results suggested that baseline suicidal ideation associated with three validated psychological scales (Rosenberg scale for self-esteem, STAI-YB Spielberger scale for trait anxiety, PHQ-9 for depression) are informative enough to identify students who will present STB at the one-year assessment.

Key strengths of this study are the large sample of students and the longitudinal design. Since there are many different paths to STB, accurate STB prediction requires the consideration of a complex combination of a large number of factors^13^. The i-Share baseline questionnaire includes a large number of variables, which enabled analyses with a large number of potential STB predictors (70 in the main analyses and 87 for the secondary analyses). Our analyses were conducted following the current recommendations and best methods for prediction analysis, especially the use of different samples for creating the predictors and then for calculating the predictive performance, which prevents the performance measures from being overfitted^10,11^. The variables identified as main predictors of STB were consistent across main and secondary analyses, suggesting robust and consistent findings. Some limitations should nevertheless be acknowledged when interpreting the results. First, the follow-up response rate (33.5%) was moderate, as is common in longitudinal studies with students^49^ and differences were observed between respondents and non-respondents in the follow-up. These differences were not major (proportions were similar) and should have a limited impact when identifying STB predictors. Nevertheless, caution is needed regarding the external validity of our results and the possibility of generalizing conclusions to all students and to all settings. Second, girls were over-represented in our sample (79%) compared to the 50–60% of female students in France^50^, and our sample might not be representative of the whole student population. Third, the self-reported questionnaires could lead to information and recall bias, particularly if participants under-reported their frequency of STB due to concerns about social desirability. However, such under-reporting is likely to be reduced by the use of an online questionnaire. Additionally, and more importantly, relying on other data (e.g., clinical assessment) would defeat our aim of finding easily assessable predictors of SBT in large university student samples. Fourth, given the adaptation of the CTQ used in this study, we could not create subsamples ‘with’ and ‘without’ childhood adversity. Thus we could not explore deeply if the identified predictors affected more individuals with childhood adversities. Finally, we could not strictly separate analyses between suicidal ideation and suicide attempts due to the small number of one-year suicide attempts in our sample even after combined the genders (n = 35).

In conclusion, we identified a parsimonious number of predictors that can be used to accurately identify students who will present STB within one-year from the predictor assessment. Pending replication of these results in other studies, these predictors may be used to develop a screening tool to be routinely used among university students. For example, a web-based screening tool could represent a promising approach for identifying students at suicide risk and to refer them to counselling and mental health services.

## Supplementary Information

Supplementary Information.

## Data Availability

The datasets used and/or analysed during the current study are available from the corresponding author on reasonable request.
