# Supplementary material for: A machine learning approach for predicting suicidal thoughts and behaviours among college students
Source: Sci Rep. 2021 Jun 15;11:11363. doi: 10.1038/s41598-021-90728-z (PMC8206419; doi:10.1038/s41598-021-90728-z)
Supplement: Supplementary file 1 — Supplementary Information. [file 41598_2021_90728_MOESM1_ESM.docx]

**SUPPLEMENTARY MATERIAL**

**A machine learning approach for predicting suicidal thoughts and behaviours among college students**

Melissa Macalli^1^*, Marie Navarro^1^*, Massimiliano Orri^1,2^, Marie Tournier^1,3^, Rodolphe Thiébaut^1,4,5^, Sylvana M. Côté^1,6^, Christophe Tzourio^1^.

*Melissa Macalli and Marie Navarro share first authorship

**Table 1.** Methods - Study design and participants

**Table 2.** Methods - Measures

**Figure 1**. Results - Secondary Analysis

**Table 1. Comparison of key variables between participants and non-participants in the follow-up**

|  | **Participants**  **(n= 5255)** | **Non-participants**  **(n= 10412)** | **P-Value** |
| --- | --- | --- | --- |
| ***Socio-demographic characteristics*** |  |  |  |
| **Age in years, mean (SD)** | 20.8 (2.6) | 21.0 (2.8) | <0.0001 |
| **Gender** |  |  | <0.0001 |
| Boys | 1099 (20.9%) | 2963 (25.9%) |  |
| Girls | 4156 (79.1%) | 7719 (74.4%) |  |
| **Opinion on resources** |  |  | <0.0001 |
| Satisfactory to very satisfactory | 4328 (82.4%) | 8050 (77.3%) |  |
| Unsatisfactory to totally unsatisfactory | 927 (17.6%) | 2360 (22.7%) |  |
| **Year of study** |  |  |  |
| First year | 2015 (38,3%) | 3880 (37.3%) | 0.1878 |
| Second year and higher | 3240 (61.7%) | 6532 (62.7%) |  |
| ***Familial characteristics*** |  |  |  |
| **Perceived parental support in childhood** |  |  | <0.0001 |
| Moderate to very high | 4789 (91.1%) | 9272 (89.1%) |  |
| Low to none | 404 (7.7%) | 943 (9.1%) |  |
| Missing | 62 (1.2%) | 196 (1.9%) |  |
| **Parental divorce** |  |  | 0.0001 |
| Yes | 1554 (29.6%) | 3311 (31.8%) |  |
| No | 3559 (67.7%) | 6730 (64.7%) |  |
| Missing | 142 (2.7%) | 369 (3.6%) |  |
| **Paternal depression history** |  |  | 0.3143 |
| Yes | 887 (16.9%) | 1766 (17.0%) |  |
| No | 3646 (69.4%) | 7125 (68.4%) |  |
| Missing | 722 (13.8%) | 1521 (14.6%) |  |
| **Maternal depression history** |  |  | 0.3967 |
| Yes | 1597 (30.4%) | 3108 (29.9%) |  |
| No | 3108 (59.1%) | 6143 (59.0%) |  |
| Missing | 550 (10.5%) | 1161 (11.2%) |  |
| ***Mental health indicators*** |  |  |  |
| **12-month suicidal ideation** |  |  | 0.0004 |
| Yes | 1151 (21.9%) | 2285 (22.0%) |  |
| No | 3950 (75.2%) | 7689 (73.9%) |  |
| Missing | 154 (2.9%) | 438 (4.2%) |  |
| **Lifetime suicide attempts** |  |  | 0.0004 |
| Yes | 298 (5.7%) | 682 (6.6%) |  |
| No | 4848 (92.3%) | 9422 (90.5%) |  |
| Missing | 109 (2.1%) | 308 (3.0%) |  |

**Table 2. Complete list of predictors included in random forests models**

| **Predictors** | **Modalities** |
| --- | --- |
| ***Socio-demographic characteristics*** |  |
| Age | in years |
| Year of study | First year |
|  | Second year and higher |
| University degree wanted | Bachelor’s degree |
|  | Master’s degree |
| Accommodation type | At parents’ home |
|  | University residence |
|  | In apartment, couple, colocation |
|  | In apartment, alone |
| Family as main sources of income | Yes |
|  | No |
| Scholarship as main sources of income | Yes |
|  | No |
| Job as main sources of income | Yes |
|  | No |
| Opinion on resources | Satisfactory to very satisfactory |
|  | Unsatisfactory to totally unsatisfactory |
| Opinion on accommodation | Satisfactory to very satisfactory |
|  | Unsatisfactory to totally unsatisfactory |
| Opinion on hobbies possibilities | Satisfactory to very satisfactory |
|  | Unsatisfactory to totally unsatisfactory |
| Opinion on social life | Satisfactory to very satisfactory |
|  | Unsatisfactory to totally unsatisfactory |
| Opinion on the relation with my parents | Satisfactory to very satisfactory |
|  | Unsatisfactory to totally unsatisfactory |
| ***Lifestyle habits*** |  |
| Bike use | Yes |
|  | No |
| Regular practice of sport | Yes |
|  | No |
| Hobbies | Yes |
|  | No |
| Sleep quality in the last 3 months | Good/Rather good/Not good or bad |
|  | Rather bad/Bad |
| Average time spent on screens/ day during the week to work | Less than 8h |
|  | More than 8h |
| Average time spent on screens/ day during the week playing games | Less than 8h |
|  | More than 8h |
| Average time spent on screens / day during the week on internet | Less than 8h |
|  | More than 8h |
| Average time spent / day during the week watching TV | Less than 8h |
|  | More than 8h |
| Average time spent / day during the week on their smartphone | Less than 8h |
|  | More than 8h |
| ***Familial characteristics*** |  |
| Having brother or sister | Yes |
|  | No |
| Parental divorce or separation | Yes |
|  | No |
| Place to live in childhood | Parents or adoptive parents |
|  | Grandparents, other family members, host family, other |
| Perceived parental support in childhood and adolescence | Moderate to very high |
|  | Low or none |
| Parental education level | University studies |
|  | No university studies |
| Brother's or sister's death | Yes |
|  | No |
| Father's death | Yes |
|  | No |
| Mother's death | Yes |
|  | No |
| Maternal depression or anxiety history | Yes |
|  | No |
| Paternal depression or anxiety history | Yes |
|  | No |
| Maternal alcohol abuse history | Yes |
|  | No |
| Paternal alcohol abuse history | Yes |
|  | No |
| Economic status in childhood | Comfortable to very comfortable |
|  | Difficult to very difficult |
| ***Physical health*** |  |
| Perceived health | Good to very good |
|  | Fair to very bad |
| Migraine | Yes |
|  | No |
| Diagnosed dyslexia | Yes |
|  | No |
| Handicap | Yes |
|  | No |
| ***Substance use*** |  |
| Tobacco use | Yes |
|  | No |
| Alcohol use | Once a week to never |
|  | Several times per week to several times per day |
| Binge drinking | Once a week to never |
|  | Several times per week to several times per day |
| Cannabis use in the last 12 months | Yes |
|  | No |
| Lifetime Ecstasy, MD, MDMA use | Yes |
|  | No |
| Lifetime amphetamine use | Yes |
|  | No |
| Lifetime cocaine use | Yes |
|  | No |
| Anxiolytic drugs use in the last 3 months | Yes |
|  | No |
| Drugs to improve concentration use in the last 3 months | Yes |
|  | No |
| Sleeping pills use in the last 3 months | Yes |
|  | No |
| Drugs to improve concentration performance use in the last 3 months | Yes |
|  | No |
| Vitamin pills or dietary supplement use in the last 3 months | Yes |
|  | No |
| ***Mental health*** |  |
| Feeling of sadness for several days in a row over the past 12 months | Yes |
|  | No |
| 12-month suicidal thoughts | Yes (occasionally or frequently) |
|  | No |
| Lifetime suicidal attempts | Yes |
|  | No |
| Lifetime traumatic event experience | Yes |
|  | No |
| Diagnosed anxiety and phobia disorders | Yes |
|  | No |
| Diagnosed obsessive-compulsive disorders | Yes |
|  | No |
| Lifetime diagnosed depression | Yes |
|  | No |
| Diagnosed ADHD | Yes |
|  | No |
| Vomiting after meal disorders | Yes |
|  | No |
| Perceived stress (PSS-4) | Score |
| ADHD (ASRS-6) | Score |
| Academic stress (Boujut and Bruchon-Scweitzer scale) | Score |
| Social support coping strategies (Boujut scale) | Score |
| Emotional coping strategies (Boujut scale) | Score |
| Festive coping strategies (Boujut scale) | Score |
| Impulsivity (BIS-11 scale) | Score |
| Aggressive behaviours (BGA scale) | Score |
| Trait-Anxiety (STAI-YB scale) | Score |
| Self-esteem (Rosenberg scale) | Score |
| Depression symptoms (PHQ-9) | Score |
| ***Childhood adversities*** |  |
| I thought that my parents wished I had never been born | Moderate to severe |
|  | No to low |
| There was nothing, I wanted to change in my family | Moderate to severe |
|  | No to low |
| People in my family hit me so hard that it left me with bruises or marks | Never or once |
|  | Yes, several times |
| I believe that I was sexually abused | Never |
|  | Rarely to several times |
| Someone threated to hurt me or tell lies about me unless I did something sexual with them | Never or once |
|  | Yes, several times |
| I had the best family in the world | Moderate to severe |
|  | No to low |
| I believe that I was physically abused | Moderate to severe |
|  | No to low |
| I believe that I was emotionally abused | Moderate to severe |
|  | No to low |
| Peer-victimization in childhood or adolescence | Moderate to severe |
|  | No to low |
| I was neglected (not enough to eat or I had to wear dirty clothes) | Moderate to severe |
|  | No to low |
| I knew that there was someone to take care of me and protect me | Moderate to severe |
|  | No to low |
| People in my family criticized me or made me look ridiculous | Moderate to severe |
|  | No to low |
| My parents were too drunk or high to take care of me | Moderate to severe |
|  | No to low |
| I was ignored or my parents made me understand that I was not important to them | Moderate to severe |
|  | No to low |
| There was someone in my family who helped me feel that I was important | Moderate to severe |
|  | No to low |
| I felt loved | Moderate to severe |
|  | No to low |
| I was treated coldly by my parents or without paying attention to me | Moderate to severe |
|  | No to low |

**Figure 1. Ranking of the importance of baseline variables in a random forests model for predicting one-year suicidal thoughts and behaviours, including childhood adversity variables, stratified by gender**

**

**
